# Model of intracranial hypertension of tumor etiology in laboratory rats

**DOI:** 10.1186/cc13649

**Published:** 2014-03-17

**Authors:** AN Kolesnikov, VI Cherniy, KA Kardash, TA Mustafin, VN Stasyuk, IV Koktishev

**Affiliations:** 1 Donetsk National Medical University of Maxim Gorky, Donetsk, Ukraine

## Introduction

The objective of this research is the creation of an experimental model of intracranial hypertension (ICH) of the tumor etiology in white nonlinear rats.

## Methods

Work was executed in the neurophysiology laboratory on 12 white nonlinear rats, weight 280 to 320 g, preselected according to criteria of bioethical rules. For modeling ICH in animals, standard anesthesia was carried out. A longitudinal section was spent in the projections of saggital suture from the frontal to occipital bone. The offensive surgical stage anesthesia showed loss of palpebral and lingual reflexes. Further, according to the stereotactic atlas coordinates, a burr hole was performed, through which sterile two-component antiallergen and depyrogenized gel in the amount of 0.025 ml was applied into the fourth ventricle with an intracerebroventricular microinjector. The injector was at an angle of 12° in order to avoid injuring vessels, its distal end entering a depth 4.5 mm in the fourth ventricle, and was installed and documented in the burr hole for measuring intracranial pressure. Monitoring during operations included: respiration, heart rate, ECG, thermometry, and control of diuresis.

## Results

In all animals the heart rate decreased to an average of 180 beats/minute during 90 minutes after the introduction of the intracerebroventricular two-component gel. Also there was a decrease in respiration rate by an average of <70 breaths/minute. Behavioral responses of animals were evaluated. The average intensity and the duration of such actions as shaking, friction, licking and carding decreased. Decline in grooming was possibly associated with breached central regulation in the brain of studied rats. Macroscopically in the preparation we observed a pronounced hyperemia of obtained materials, which may indirectly indicate the syndrome of ICH. Survival of the animals was: 3 days - 100%, 7 days - 100%, 14 days - 92%, 21 days - 83%.

## Conclusion

The main result of this work can be considered creation of a successful and workable model of ICH. The developed model is the testing ground to assess especially anesthesia protection in patients with the syndrome of ICH including tumor etiology and has no analogs.

